# Privacy and ethical challenges of the Amelogenin sex test in forensic paternity/kinship analysis: Insights from a 13-year case history

**DOI:** 10.1016/j.fsisyn.2023.100440

**Published:** 2023-09-29

**Authors:** Alessandro Gabriele, Elena Chierto, Sarah Gino, Serena Inturri, Serena Aneli, Carlo Robino

**Affiliations:** a Department of Public Health Sciences and Pediatrics, University of Turin, C.so Galileo Galilei 22, 10126, Turin, Italy; b Department of Health Sciences, University of Eastern Piedmont, Via Solaroli 17, 28100, Novara, Italy

**Keywords:** Incidental findings, Amelogenin test, Forensic genetics, Differences/disorders of sex development (DSD), Klinefelter syndrome

## Abstract

The Amelogenin sex test included in forensic DNA typing kits has the potential to identify congenital conditions such as differences/disorders of sex development (DSD). It can also reveal mismatches between genotypic sex and gender marker in identity documents of transgender persons who obtained legal gender recognition.

In a 13-year case history of paternity/kinship tests, involving n = 962 females and n = 1001 males, two mismatches between Amelogenin sex test (male) and gender marker (female), and three cases of chromosomal DSD (Klinefelter syndrome) were observed.

The concrete risk of observing Amelogenin anomalies, their potential causes, and the context in which they occur (forensic, i.e. non-medical) mean that laboratory operators are called to strike a complex balance between privacy interests and individual health rights when providing preliminary information and reporting Amelogenin incidental findings. This case history argues for the need of a more responsible approach towards the Amelogenin sex test in the forensic community.

## 1 Introduction

Amelogenin is a gene encoding for a matrix protein of tooth enamel located in the pseudoautosomal region of the human sex chromosomes [1]. Copies of the Amelogenin gene on the p22.1-p22.3 region of the X chromosome (AMELX) and the p11.2 region of the Y chromosome (AMELY), while highly homologous [2], carry several sequence differences that enable sex typing of forensic samples. The Amelogenin sex test, first described in 1993, takes advantage of a 6bp deletion within the first intron of AMELX [3], allowing electrophoretic separation of X- and Y-specific PCR products.

Determination of sex chromosome genotype in human stains of unknown origin found at the crime scene and in unidentified human remains can be highly beneficial in the preliminary phase of forensic investigations. It was therefore understandable for Amelogenin-specific primers to be included from the very beginning in short tandem repeat (STR) multiplex PCR amplification systems developed for forensic purposes [4]. Since its introduction, several limitations of the Amelogenin sex test have emerged, in particular, AMELY amplification failure due to a large deletion on the short arm of the human Y chromosome was found to be rather common, especially in the Indian subcontinent with about 2% of males testing AMELY-negative [5]. The consequent relevant risk of investigative errors prompted the incorporation of integrative sex markers, such as the Y-STR DYS391 and a Y-chromosomal insertion/deletion marker (Yindel), in several recently introduced commercial STR kits [6]. Nevertheless, almost all STR profiling systems used nowadays in forensics still include Amelogenin as sex marker.

Commercial forensic STR kits are used interchangeably in casework (analysis of unknown stains and unidentified human remains) and in genotyping of reference samples for DNA profile comparisons, criminal DNA databases, and paternity/kinship testing. For the latter applications, the presence of Amelogenin in DNA typing kits means that information regarding the sex-chromosome constitution of the tested individuals is obtained, possibly revealing a wide array of congenital conditions, cumulatively known as differences/disorders of sex development (DSD), associated with heterogeneous health risks and infertility [7]. DSD include atypical development of chromosomal, gonadal or phenotypic sex grouped into three categories according to basic genetic characterization, namely: 46,XY DSD (phenotypic female with a male genotype); 46,XX DSD (phenotypic male with a female genotype); and sex chromosomal DSD (aneuploidies) [8]. Suspicion of sex chromosomal DSD may rise as a consequence of the fact that capillary electrophoresis detection of dye-labelled STR and Amelogenin PCR products provides quantitative information, derived from measurement of peak heights in electropherograms. In forensic investigations, analysis of AMELX/AMELY peak height ratio aids the interpretation of male-female DNA mixtures [9]. However, the same quantitative information has been routinely employed in prenatal diagnosis to detect common chromosome aneuploidies by means of quantitative fluorescence multiplex PCR (QF-PCR), targeting selected STRs and the Amelogenin gene [10]. In particular, many numerical sex chromosomal disorders are readily identified when asymmetry is observed between X- and Y-specific peaks within the Amelogenin locus [11].

Operators should be aware of the concrete chance to incidentally detect DSD during a forensic test and detailed policies should be developed to convey preliminary information to tested individuals and define how unexpected results should be communicated. Several reports of incidental findings at the Amelogenin locus can be found in the forensic literature [[12], [13], [14], [15]], and authors have warned against the consequent risk of misleading information in criminal identification [16]. On the contrary, little or no attention was paid so far to Amelogenin in the context of the emerging debate on the management of unexpected findings in forensic investigations [17,18]. Concerns related to indiscriminate use of sensitive genetic information for the inference of ancestry and externally visible characteristics, now made easier by the introduction of next generation sequencing (NGS) technologies, prompted a fruitful scientific debate [19] and lead to the adoption of specific regulations in a few European countries [20]. However, only Sweden seems to have caught the potential of sensitive information carried by the Amelogenin locus, by forbidding the inclusion of sex as determined by Amelogenin in the national DNA database [21]. In the UK National DNA Database, by contrast, it is current practice that no feedback about results potentially providing information on the individual's health (including Amelogenin) is returned when DNA reference profiles are obtained from staff for inclusion on elimination databases [22]. Commercial suppliers of NGS-based forensic DNA typing reagents have proven sensitive to the matter, by providing separate primer mixes for identity and phenotypic applications available within the same NGS kit [23]. However, even in such cases, it was chosen to include Amelogenin, though strictly related to phenotype, in the “identity” primer mix.

As of 2022, most Council of Europe states guarantee transgender people, who want to be legally recognized as a man or a woman, a juridical and/or administrative path to change their legal gender (“gender marker”) on identity documents [24,25]. In particular, legal gender recognition in Italy is affirmed by Law n° 164/1982 “Rules concerning the Rectification of Sex-Attribution”. As a consequence, the Amelogenin sex test -by revealing a possible change of gender marker-may also violate the right to privacy when performed without the prior, free, and informed consent of the person concerned [26].

Presently, no specific recommendations/guidelines regarding preliminary information about the Amelogenin sex test, including the option to choose to receive unexpected results, are offered to the forensic genetics community by relevant scientific organizations, such as the International Society for Forensic Genetics, or international advisory groups like the European Network of Forensic Science Institutes.

To stimulate discussion on the sensitive topic of incidental Amelogenin findings, we have revised our recent laboratory case history of paternity/kinship (PK) tests (years 2009-2021). PK cases in which unexpected results in the Amelogenin sex test were observed and their practical and ethical challenges are discussed in the context of the Italian and European legal framework.

## 2 Materials and methods

Retrospective analysis of paternity data for the present study was authorized by University's internal review board (Protocol nr. 0202730 13/04/2022). Between 2009 and 2021, a total of 1963 individuals underwent PK investigations including tests directed by a civil court (PKC) (n = 970), commissioned by private reasons (PKP) (n = 861), and immigration cases (PKI) i.e., voluntary testing of petitioners missing adequate documentation to prove family relationships performed upon request of local migration authorities (n = 132).

All tested individuals were identified by personal documents (identity card, passport, residency permit, etc.) and gave their informed consent prior to DNA testing. In case of minors or incapacitated persons, preliminary authorization by parents or the legal guardian was obtained. Preliminary information was provided by medical doctors with a background in forensic genetics. Tested subjects (parents and legal guardians in case of minors/incapacitated individuals) were informed that according to the Italian legislation on data protection and privacy “General Authorisation for the Processing of Genetic Data” [27], prior to genetic testing “data subject shall have to state whether he/she wishes to be informed of the findings of the test/research, including unexpected findings concerning him/her where such findings are factually and directly beneficial to the data subject in terms of treatment, prevention, and/or awareness of reproductive choices”. In compliance with this, they were made aware that the Amelogenin test included in STR typing kits, by providing information on the constitution of sex chromosomes, could raise the suspicion of congenital conditions. They were then offered an opt-in, opt-out option with the specification that, in case of an Amelogenin incidental finding, this would not be explicitly highlighted in the PK test final report and exclusively communicated to the affected individual or the entitled subjects. Case history also included cadaver samples and/or archival samples from deceased individuals (deficiency cases) which were analyzed upon court order.

Across the considered period, different DNA extraction protocols, chosen depending on sample type, and several autosomal STR typing kits including Amelogenin were employed according to manufacturer's instructions. They are summarized in Fig. S1. STR genotyping was performed using the ABI Prism 310 Genetic Analyzer (Applied Biosystems) between 2009 and 2018, and subsequently with the SeqStudio Genetic Analyzer (ThermoFisher Scientific). Data analysis was carried out with Genotyper software (Applied Biosystems) until 2012 and thereafter with GeneMapper software (ThermoFisher Scientific).

An Amelogenin test result was defined as unexpected when: 1) an Amelogenin genotype discordant with gender marker reported on the identification document (hereafter simply indicated as “gender”) was observed 2) peak height ratio between AMELX and AMELY deviated of at least+/-3 SD from the mean value observed in the internal validation performed for the specific STR kit. AMELX:AMELY imbalance was not evaluated in samples supposedly affected by DNA degradation (i.e. bone, formalin fixed paraffin embedded tissues, etc.). In all cases, unexpected Amelogenin observations needed to be confirmed on an independent replicate sample typed with a different STR kit.

In the PK case involving subject PKC-1, described in detail in the Results section, likelihood ratio (LR) calculations for autosomal STRs were performed with Familias software [28], using allele frequency data observed in the Italian population [29,30], with the exception of locus SE33 for which general European data [31] were considered. A correction Fst = 0.01 was applied. For X-STRs (Investigator Argus X-12, Qiagen), LR value was estimated with FamlinkX software [32] and “cluster” approach, using haplotype diversity data for the Italian population [33] and recombination rates reported by Nothnagel et al. [34].

Other statistical analyses (pairwise Wilcoxon Rank Sum test, chi-square test and multimode computation) and graphical representations were conducted using R version 4.1.3 [35].

## 3 Results

### 3.1 General characteristics of tested subjects

**Fig. 1:**
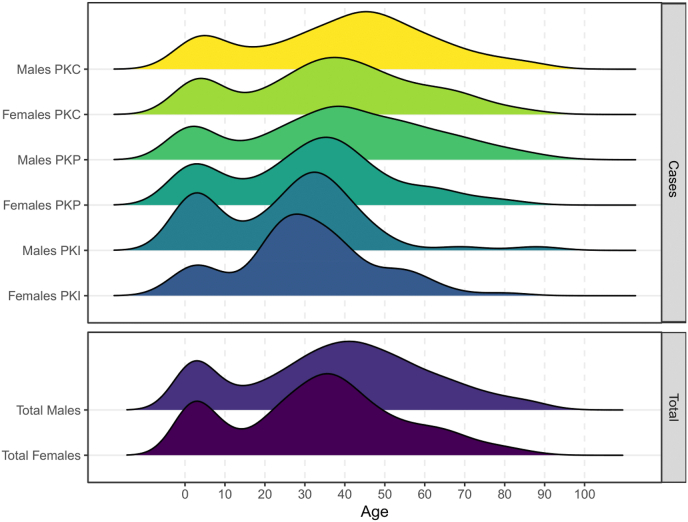
Density plot of the age distribution of tested individuals by category of PK test (PKC: court-directed test; PKP: private test; PKI; immigration test) and gender.

Tested individuals included 51% of males (n = 1001) and 49% of females (n = 962), according to gender. Age distributions of subjects undergoing PK tests were typically bimodal in both genders and all three PK categories (Fig. 1, Table S1**)**. Statistically significant differences were observed between the three categories (males and females combined), with subjects undergoing PKC tests having an older age and those involved in PKI tests being the youngest (p-values lower than 0.0001 for all comparisons).

Most individuals undergoing PKI tests (88.6%) were of non-Italian citizenship. However, it could be seen that the percentage of ubjects with non-Italian citizenship was not negligible also in PKC and PKP tests (13.8% and 17.2%, respectively).

### 3.2 Stated preferences to know or not to know about anticipated incidental findings

**Fig. 2:**
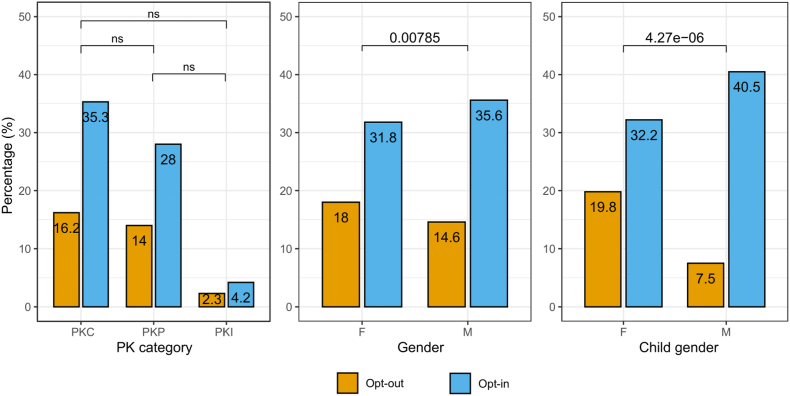
Options regarding the disclosure of Amelogenin incidental findings expressed by adult tested subjects divided by: category of PK test; gender; gender of tested minor children. Chi-square p-values are reported within each panel. ns: not significant.

Decisions regarding the option to receive unexpected Amelogenin findings are summarized in Fig. 2. No significant differences were observed in preferences between tested individuals undergoing different categories of PK test, with about two thirds of subjects opting-in for information in each category. On the other hand, sharp differences were seen when comparing preferences between genders, with a significantly higher percentage of male individuals and parents of male minors opting-in for information.

### 3.3 Incidental Amelogenin findings

In our case history, we observed two female adult subjects, i.e. individuals carrying identity documents with female gender marker, who displayed a balanced AMELX/AMELY genotype at the Amelogenin locus in two independent replicate analyses of buccal swabs using different STR typing kits. Both cases were recorded in 2009. One observation occurred within the context of a PKI case in a 27-year-old woman (alleged sister of the applicant), and the other in a PKP test in which the subject displaying sex genotype/phenotype discrepancy was the alleged daughter (42-year-old). The causes underlying genotype/phenotype mismatches were not further investigated, due to the specific and limited purpose of PK tests. According to set preferences, the 46, XY female from PKI was informed of the unexpected finding. While lamenting primary amenorrhea, she was reportedly unaware of an underlying genetic condition and was therefore forwarded to the Medical Genetics Unit of the local University Hospital. On the other hand, the unexpected Amelogenin finding was not explicitly shared with tested individual in the PKP case.

Three male individuals displaying anomalous imbalance between AMELX and AMELY were also observed in our case history. All cases involved adult male samples subjected to PKC testing, identified hereafter as PKC-1 (year of observation 2015), PKC-2 (year of observation 2016) and PKC-3 (year of observation 2020). As indicated in Table 1, all three individuals had about two-fold AMELX signal compared to AMELY suggesting a 47,XXY karyotype (Klinefelter syndrome, KS).

**Table 1:** STR-kit specific observed and expected peak height ratios in three male individuals displaying anomalous imbalance between AMELX and AMELY.

Case	STR kit	Observed AMELX:AMELY ratio	Expected AMELX:AMELY ratio (±SD)
PKC-1	AmpF*l*STR Identifiler Plus	2.524	1.010 ± 0.156
	Investigator ESSplex SE	1.456	0.983 ± 0.098
PKC-2	AmpF*l*STR Identifiler Plus	2.274	1.010 ± 0.156
	Investigator ESSplex SE	1.589	0.983 ± 0.098
PKC-3	PowerPlex ESX 17 Fast	1.728	0.919 ± 0.058
	AmpF*l*STR Identifiler Plus	2.018	1.010 ± 0.156

In cases PKC-1 (43-year-old male) and PKC-2 (51-year-old male), the involved subjects had opted-in for information on Amelogenin incidental findings of which both, apparently, were previously unaware of at the time of communication. Consequently, they were both offered a consultation at the University Hospital Medical Genetics Unit. In case PKC-3, the posthumous discovery of a clinical diagnosis of KS in the late father prompted his adult offspring to file a disclaimer of paternity. Notably, in this case, the judge allowed the use of archival DNA, previously collected for medical genetic testing, as a reference sample of the deceased father in order to avoid the exhumation of the body.

**Fig. 3:**
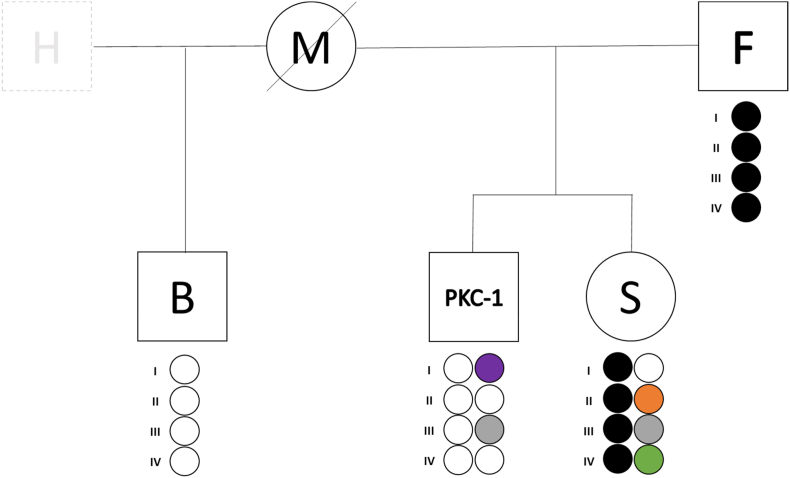
Family tree of PKC-1, including his deceased mother M, father F, half-brother B (son on M and her husband H), and alleged full-sister S (daughter of F and M). Small circles represent the four X-STR linkage groups. Maternal haplotypes as derived from the analysis of B are shown in white. Colored circles correspond to additional maternal haplotypes identified in PKC-1 and S. Paternal haplotypes of S are shown in black.

Of particular relevance in our case history was KS case PKC-1, identified in the context of a court-ordered maternity test which, as depicted in Fig. 3, also involved: PKC-1's mother “M" (deceased) and father “F"; PKC-1's half-brother “B" (son of M and her husband “H”); PKC-1's alleged full-sister “S" (daughter of M and F). The litigation arose from the fact that, according to S's claim, M had been unable to recognize her at birth, since S was conceived with F, a man other than M's husband H. At that time (1960's) the Italian legislation still did not contemplate divorce and prevented members of married couples to recognize children born outside of wedlock. Such prohibition remained in effect until 1970, when the Italian law on divorce was introduced (Law n° 898/1970) [36]. Though now allowed to recognize S, M and F had omitted to do so, whereas they subsequently recognized S's younger brother PKC-1 when he was born. This led to a maternity suit after the death of M and to the following court order for a DNA test.

Since the body of M had been cremated and no archival reference sample was available, PKC-1 and B were requested to undergo DNA testing in order to reconstruct their mother's genotype and compare it with maternal alleles of S, identified through comparison with her biological father F. Analysis of 21 STRs (the combination of AmpF*l*STR Identifiler Plus and ESSplex SE Plus kits), which represented the most extended autosomal STR panel available in the laboratory at the time of testing, led to a LR of the genotypes given the hypothesis of full sibship between PKC-1 and S, rather than half-sibship, equal to 6.9 x 10^3^. This value was below the minimal threshold (LR > 10^4^) requested in recommendations drawn by the Italian working group of the International Society for Forensic Genetics (Genetisti Forensi Italiani, GEFI) [37] and the Italian Society of Human Genetics (SIGU) [38] to consider the relationship “practically proven” in test reports.

Since the inspection of Amelogenin locus had evidenced a likely KS in PKC-1, he was informed of the result according to set preferences and about the possibility to use his extra X chromosome to better characterize the genotype of M, thus possibly increasing the final LR. PKC-1 consented to further analysis, so that in this exceptional case the incidental finding of KS was not only personally communicated to the interested subject, but also included and justified in the final report for the court.

Analysis of X-STRs included in the Investigator Argus X-12 kit showed that the extra X chromosome of PKC-1 was evidently of maternal origin (Fig. 3). Through comparison with B and F, it was possible to infer the maternal X-STR haplotypes of PKC-1 and S, respectively. It was then evident that S, PKC-1 and B carried the same maternal X-STR haplotype at linkage group (LG) I and that S and PKC-1 also shared an identical maternal haplotype in LG III. LR calculations were performed with the software FamlinkX, applying the following expedients: PKC-1 was considered as a diploid female individual; paternity of PKC-1 by F was disregarded in competing pedigrees, given that both X chromosomes of PKC-1 were of maternal origin. Calculations lead to a LR = 5.1 x 10^2^ for observed X-STR genotypes given the hypothesis that PKC-1 and S had the same mother rather than different mothers. This result could be combined [39] with LR obtained for autosomal STR loci, thus leading to a cumulative LR that exceeded the minimal threshold indicated in relevant recommendations by national scientific societies.

Although final ruling in PK cases must stem from the combined evaluation of circumstantial and genetic data, it is common for the judge in complex cases with lack of additional documentation to strongly rely on the results of DNA testing. Therefore, it can be speculated that, in the present case, refusal by PKC-1 to be informed regarding unexpected findings at the Amelogenin locus could have negatively impacted the rightful claim of S.

## 4 Discussion

Our results show how, even in a limited PK case history, incidental findings at the Amelogenin locus are expected to occur and must therefore be dealt with. Those at the Amelogenin locus are not the only health-related which can be observed during standard STR typing. Trisomy-21, Trisomy-18 and Trisomy-13, for instance, give raise to atypical and easily identified allelic patterns [40]. However, nearly all trisomic patients receive a diagnosis long before forensic DNA testing, and their condition is generally known to others due to the severity of the phenotype [41]. By contrast, some individuals may be completely unaware of anomalies in their sex-chromosome constitution revealed by the Amelogenin test.

Many individuals with a DSD are recognized at birth, through the observation of ambiguous genitalia, or even in the prenatal period thanks to karyotyping and ultrasound evaluation [42]. For the commonest cause of 46,XX DSD (congenital adrenal hyperplasia due to 21-hydroxylase deficiency) neonatal biochemical screening programs have been introduced worldwide, effectively improving time to diagnosis [43]. However, some types of DSD can still elude early diagnosis. This is especially true of areas of the world where prenatal diagnosis is still largely unavailable [44], making immigration DNA tests potentially prone to incidental discoveries. As for 46,XY females, the most common cause is the androgen insensitivity syndrome (AIS), followed by gonadal dysgenesis [45]. Incidence of AIS is reported to be up to 5 per 100,000 births, that of gonadal dysgenesis 1 per 80,000 births [45]. While 25% of 46,XY DSD females are identified by the age of 3 years, most cases defy diagnosis and may become evident later (median age at diagnosis 14 years, with reported diagnosis as late as 67 years [45]) following observation of absent, incomplete or delayed puberty, virilization or gynecomastia, or medical investigations undertaken in case of infertility or gonadal tumors [46].

As regards the main sex chromosome DSD (KS), in absence of prenatal karyotype testing, early to middle adulthood represents the typical age at diagnosis [47]. KS is by large the most common cause of male hypogonadism and, because of the extremely variable phenotype, it is estimated that 50 to 75% of KS individuals never obtain a correct diagnosis in their life, with median age at diagnosis of 27 [48]. A recent study conducted on over 200,000 men of European ancestry aged 40 to 70 years from the UK Biobank showed that only 23% of KS cases identified by genotyping array or exome sequencing were aware of their condition [49]. The incidence rate of KS in our male sample (about 1 in 300) was slightly higher than that currently estimated in the general population (9-22:10,000 births) [49,50]. This was not unexpected in a case history of PK tests, due to the fact that KS is a cause of infertility and several PK tests are conducted to exclude paternity.

Since the risk of incidental findings at the Amelogenin locus is far from negligible, particularly in the specific context of PK testing, the first question to be addressed is then that of the validity of forensic Amelogenin in accurately identifying DSD. In QF-PCR prenatal diagnosis, information derived from the Amelogenin locus in combination with a Y chromosome marker has been routinely used to assess fetal sex and identify sex chromosome aneuploidies [11]. Current forensic STR typing kits including Amelogenin can be potentially valid for the same purpose [[12], [13], [14], [15], [16]]. In some instances, incidental identification of KS even proved relevant for the interpretation of genetic data in forensic investigations [51,52]. Management of genetic incidental findings, especially in the fast-growing area of clinical whole-genome and whole-exome sequencing, has been the subject of a long-standing debate [[53], [54], [55]]. There is general agreement that, according to the ethical principle of beneficence, only findings with a high threshold of clinical utility should be reported. This can be done either by defining a set list of reportable conditions, as advocated by the American College of Medical Genetics and Genomics [56], or by leaving to professionals the final decision on what to report based on genetic penetrance and actionability, as suggested by the European Society of Human Genetics [57]. Recommendations from interdisciplinary working groups and professional bodies are that, prior to testing, competent adults should be given the option to receive (or decline) information about incidental findings unrelated to the primary test indication [[58], [59], [60]]. This principle of patient autonomy has been also adopted by the Italian “General Authorisation for the Processing of Genetic Data” [27].

Clear and effective preliminary genetic testing information may be hampered by language barriers. In that case, preliminary information to non-Italian speakers, or Italian-speaking foreign nationals who made a request, was always conveyed in a language understandable to the tested subjects. When applicants understood and spoke English and/or French and/or German, information was provided directly by laboratory health professionals. In all other cases, assistance by an interpreter and/or cultural mediator was guaranteed. Our results, showing that subjects who underwent PKC, PKP, and PKI tests responded similarly regarding the possibility to opt-in or opt-out about Amelogenin incidental findings, contradict the idea that PKI test applicants will have little interest in additional information, even if potentially relevant for their health, since they may perceive the DNA test simply as one more bureaucratic requirement in a long and exhausting administrative process [61].

Meaningful choices do not only depend on the way information is conveyed, but also on its content. What should be carefully considered here is the value of disclosing unexpected Amelogenin results. A balance should be achieved between: beneficence, intended as clinical actionability and the opportunity for tested subjects to make meaningful life and reproductive decisions; duty of non-maleficence, i.e. the risk of providing information that results unclear and a possible source of emotional distress.

Strictly speaking, the role of laboratory personnel in charge of PK testing is to clearly outline the possibility of DSD discovery, leaving further explanations and follow-up to clinical geneticists and endocrinologists, in case the tested subjects had opted-in about Amelogenin incidental findings and those occurred. However, it is common that tested subjects, in order to take a knowledgeable decision, may ask the PK testing operators for further information about DSD at the time of sample collection.

The broad term DSD encompasses a complex array of conditions, some of which of borderline clinical significance. The term DSD itself, while promptly adopted by medical professionals, has been contested by several self-advocacy groups, who prefer the term “intersex” [42]. They underline the negative connotations of DSD, including the stigma connected to the term “disorder”, the implication that intersex bodies should be medically “fixed” [62], and the simplistic assumption that innate variation of sex characteristics can be framed and addressed in terms of sexual orientation or gender identity [63]. Hence the need for a shift of focus from treatment to counselling and attendance, in particular in the case of children/adolescents and their families, having self-acceptance and improvement of quality of life as final aims [64].

The wide phenotypic spectrum of DSD with all their complex medical and psychological aspects can be hardly summarized in a synthetic description. As reviewed in Gravholt et al. [48] KS, the commonest type of DSD and the most prone to incidental findings, besides hypergonadotropic hypogonadism, testosterone deficiency and infertility, may also include cognitive impairment and increased risk of type 2 diabetes, cardiovascular disease, breast cancer, and extragonadal germ cell tumors, leading to significantly lower self-perceived quality of life and higher morbidity and mortality rates compared to the general population [48]. Several measures can be enacted to prevent severe disease and improve quality of life in KS [65], thus justifying the value of disclosure of unexpected results to individuals who opted-in for information [66]. Such measures may include: language therapy and developmental support in childhood; treatment with testosterone starting from puberty, when hypogonadism is already present, and then in adult life to reduce fat mass and improve muscle strength, bone density, libido and mood; enhanced surveillance for metabolic syndrome, cardiovascular and male breast cancer.

Our results showed that, while there were no significant differences in opt-in opt-out options between tested individuals undergoing different categories of PK test, striking differences were seen between genders, with a significantly higher percentage of male individuals and parents of male minors opting-in for information. This observation can be partly explained with the propensity of laboratory personnel providing preliminary information about the Amelogenin sex test to specifically focus on KS, due to higher incidence and increased risk of late diagnosis, compared to other DSD. However, we cannot exclude that the scarcity of opt-in preferences observed in parents of female minors in particular, compared to their male counterparts, may also be the consequence of ineffective communication about 46,XY DSD. Forensic laboratories usually employ personnel with a varied background in biomedical sciences [67]. In this specific case history it was medical doctors with a background in forensic sciences and forensic genetics. However, forensic genetics education and training programs generally do not provide the full range of expertise -combining nedical genetics, psychology, bioethics and communication skills-which is needed to manage the complex issue of a DSD/intersex diagnosis [64]. This could affect the ability of forensic experts to give detailed and complete information on the personal and health implications of variant sex characteristics potentially revealed by the Amelogenin test, thus limiting the opportunity for tested subjects to make meaningful choices about incidental findings.

This problem is connected with the ambiguous status of PK testing and the debated question of whether it should be considered a medical test [68]. According to the Italian Code of Medical Ethics (Art. 35), informed consent to any medical act must always be obtained by a registered medical doctor/physician. Specific legislations concerning genetic testing in other European countries, e.g. German Gendiagnostikgesetz (Genetic Diagnosis Act) require individualised medical supervision for all genetic tests with important implications for the health of the person concerned or of members of their family, or for choices concerning procreation [69]. It is apparent that incidental results of Amelogenin testing will have health implications or give relevant information for future reproductive choices. Therefore, direct involvement or supervision by medical personnel or health professionals with qualifications in genetics should be recommended at the time of preliminary information and communication of results in case of incidental findings [70].

This option is obviously precluded in the case of “direct to consumer” (DTC) PKP tests. Besides the general concerns raised by DTC genetic testing in terms of adequacy of consent obtained from users [71], it should be stressed that, according to a recent survey, even basic preliminary information about unexpected or sensitive results is generally lacking in DTC tests, with just a minority of providers vaguely mentioning this possibility, and no specific reference to Amelogenin [72].

Often tested subjects will be aware in advance of possible discrepancies between Amelogenin results and gender marker reported in identity documents. This will give them the opportunity to state preferences regarding information on anomalies of Amelogenin sex-typing results in a way that is most respectful of privacy. However, the reporting of results of the DNA test may still pose some challenges. It is the case of adults with DSD/intersex and of transgender persons who intentionally opted for a sex different from that assigned at birth through legal gender recognition. While no specific data are currently available for Italy, in the nine European countries where gender recognition procedures are the most straightforward, i.e. based on the self-declared gender identity of the person, the actual prevalence of individuals who were recognized legal gender recognition ranges between 1:1000 (Iceland) and 1:10,000 (Portugal) [73]. It was also estimated that over 300,000 transgender persons who had obtained legal gender recognition lived in the USA in 2015 [74]. In such cases, inadequate advanced information and improper management of Amelogenin sex test results could violate the privacy of individuals and, consequently, jeopardize the positive feelings of security, self-recognition and reduction of psychological burden generally associated with the achievement of legal gender recognition [75].

According to the GEFI recommendations on PK testing [37], full reports should be returned to entitled individuals (tested adults, legal representatives of minor/incapacitated subjects, as well as judges, lawyers and experts appointed by parties in court-directed cases) including genotyping tables and electropherograms. Only basic knowledge of human genetics is required to interpret Amelogenin results included in genotyping tables and electropherograms. As a result, tested subjects are forcibly exposed to information regarding their sex-chromosome consitution when receiving full reports, even in the case they had opted-out. Similarly, full reports enable third-parties to obtain sensitive information about other individuals. These may include specific congenital conditions, like KS, or a mismatch between genotypic sex and gender marker. For this reason, in spite of [37], we have adopted the general policy to exclude Amelogenin test results from genotyping data summarized in tabular format and attached to final reports. This can be pleonastic in case of KS, since in PK testing only the labels of observed electrophoretic peaks are normally exported in genotyping tables, not their relative dosage or height in relative fluorescent units (RFUs), so that the Amelogenin genotype of an individual with KS will be simply indicated as X-Y. However, this mode of reporting can guarantee confidentiality when 46,XY DSD, 46,XX DSD or transgender persons are involved. Systematic editing of electropherograms to conceal Amelogenin peak profiles with RFU values is more questionable, since it may arise suspicion of manipulation of the graphical output of the analysis. In addition, specific editing of electropherograms only in presence of unexpected Amelogenin findings may be taken as indirect evidence of an anomalous sex-typing result.

Another problem applies to the typical forensic circumstance in which one of the tested subjects is deceased, with no indications on how to manage their genetic data and share documentation with third-parties entitled to receive a copy of the report. There is now general agreement that protection of “private life” granted by Article 8 of the European Convention of Human Rights (ECHR) encompasses the right to know one's ascendants, as outlined in ECHR decision Jaggi vs Switzerland (2008) [76], making court directed use of archival biological samples in PKC cases acceptable, also according to Italian guidelines for paternity analysis [37]. In the previously described case PKC-3, the diagnosis of KS was known in advance to all parties involved, but it cannot be excluded that the detection of Amelogenin anomalies in archival samples may occur unexpectedly. This information will not be relevant for the assessment of paternity but may carry significant implications for subjects who are party to the dispute, e.g. legal children of the deceased who were not directly involved in the PK test but are entitled to receive a copy of the final report.

A possible, radical solution would be the introduction of forensic autosomal STR typing kits free of Amelogenin primers, explicitly designed for PK and reference sample testing, including criminal DNA databanking. For such applications, Amelogenin can be useful as an internal control to identify possible mix-up of DNA samples, caused by laboratory mismanagement or even intentional fraud enacted by tested subjects. Other than that, the removal of Amelogenin neither impacts the outcome of laboratory analysis, nor can determine loss of information, excluding exceptional cases such as the one previously described (PKC-1). Impossibility to define Amelogenin genotype can effectively safeguard the privacy of tested individuals. By exonerating laboratory operators from the need of delving into descriptions of DSD and their potential impact on health and future quality of life, it also prevents the risk of inappropriate and misleading information. Such a danger is concrete in the case of subjects undergoing a forensic test, generally perceived as non-medical, who may not be adequately prepared to decide about health-related issues potentially stemming from the analysis. On the other hand, it must be underlined that this option would close a window of opportunity for unaware carriers of DSD by delaying diagnosis, meaningful choices regarding treatment and provision of specialized care and social welfare assistance benefits.

## 5 Conclusions

It is evident that the widespread use of Amelogenin in forensic testing can lead to potential violations of privacy and incidental discovery of genetic conditions associated with health and reproductive issues. Circumstances leading to anomalous Amelogenin results are altogether rare. However, the enormous amount of STR typing tests performed nowadays for paternity assessment and for the inclusion of reference samples in national DNA databases means that even laboratories with low-to medium throughput casework must be prepared for such events.

The case history here reported highlights how incidental Amelogenin findings may represent a complex challenge for laboratory operators. Not informing in advance the tested subjects about the Amelogenin sex test, its possible outcomes, and the available options regarding the communication of unexpected results should be considered unethical and even unlawful within specific normative frameworks, such as the one defined by the Italian legislation on data protection and privacy. Adoption of Amelogenin-free STR typing kits could be a resolution, but such kits are not commercially available at this moment. Moreover, it would deny unaware carriers of DSD a chance of diagnosis.

So far, the “Amelogenin issue” has been largely overlooked by governmental, academic and private laboratories performing DNA tests on request of judicial and immigration authorities or commissioned by commercial clients, including DTC tests. As it happened in the case of incidental findings in clinical genomic testing, it would be highly desirable to encourage national and international discussion on this topic. The final aim could be to set standardized guidelines on preliminary information and reporting of results to tested/entitled subjects, so that a balance is achieved between privacy and individual health rights.

## Funding

This research did not receive any specific grant from funding agencies in the public, commercial, or not-for-profit sectors.

## Declaration of competing interest

The authors declare that they have no known competing financial interests or personal relationships that could have appeared to influence the work reported in this paper.
