# Supplementary material for: Privacy and ethical challenges of the Amelogenin sex test in forensic paternity/kinship analysis: Insights from a 13-year case history
Source: Forensic Sci Int Synerg. 2023 Sep 29;7:100440. doi: 10.1016/j.fsisyn.2023.100440 (PMC10568343; doi:10.1016/j.fsisyn.2023.100440)
Supplement: Multimedia component 1 [file mmc1.pdf]

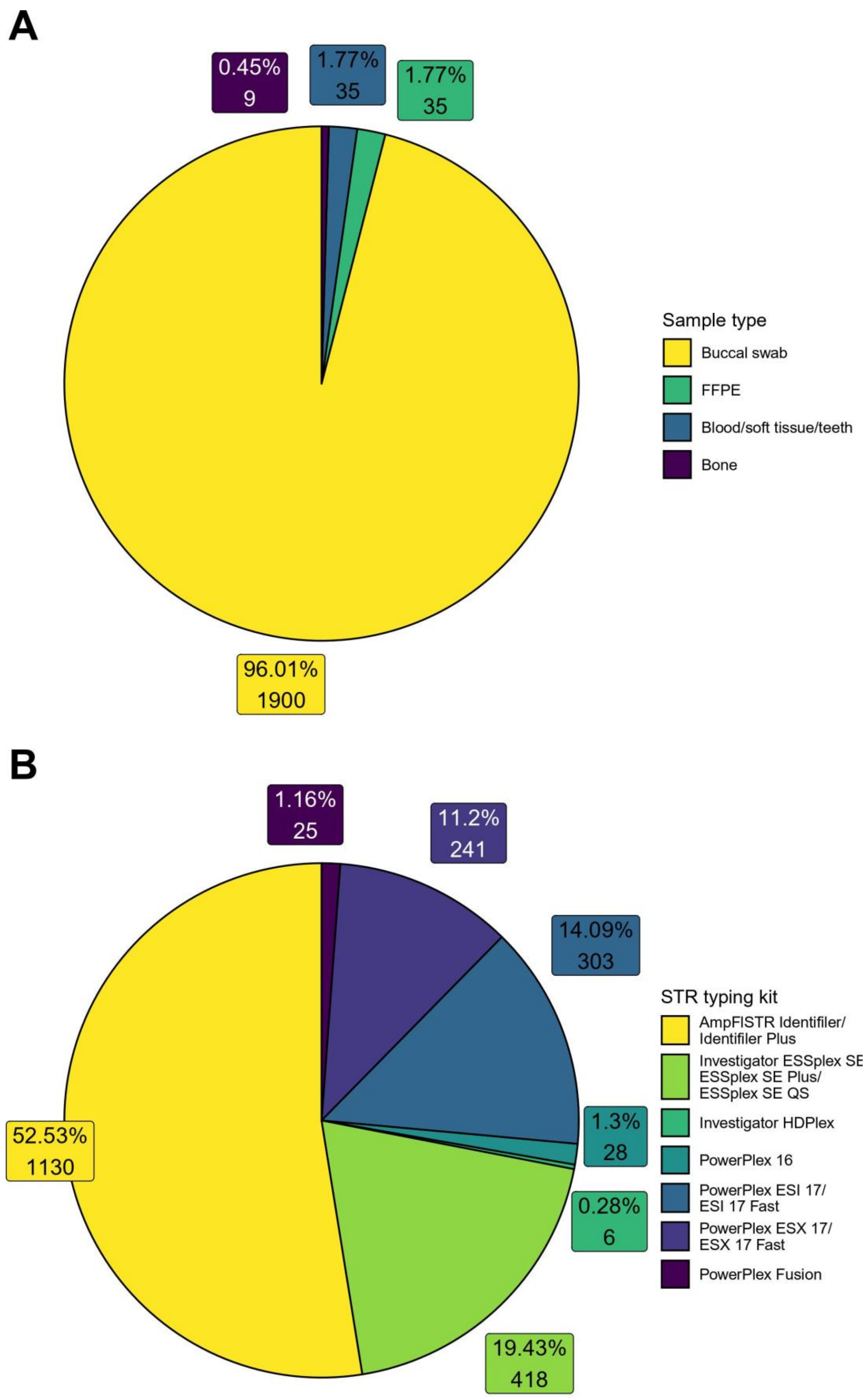

Figure S1. Absolute numbers and percentages of PK tests performed in our laboratory divided according to: A) DNA extraction protocols, chosen depending on sample type (FFPE=formalin fixed paraffine embedded tissue); B) autosomal STR typing kits. It must be noted that in some PK cases different sample types and/or multiple STR kits were tested in the same individual.

	**MODE 1**	**MODE 2**
Males PKC	3.6	44.6
Females PKC	3.2	36.0
Males PKP	1.4	38.3
Females PKP	2.3	35.6
Males PKI	5.6	32.1
Females PKI	3.2	27.9
Total Males	2.1	42.2
Total Females	2.0	36.0

Table S1. Inferred age distribution modes of individuals involved in PK tests.
